# Biographical: D. O. Hawxhurst

**Published:** 1882-03

**Authors:** 


### BIOGRAPHICAL.

D. O. Hawxhurst M.D. and D.C., of Battle Creek, Michigan, died of small-pox, at Paris, France, February 16, 1882, in the fortieth year of his age.

Dr. Hawxhurst was born in Oxford, Oakland County, Michigan. His early education was directed by his mother. From eight to twelve he attended the common schools, and then spent three years in Bedford Seminary, Michigan. There his natural taste for anatomy, chemistry and physics found opportunity for cultivation. He spent a year and a half under the tutorship of a geologist, gathering specimens and occasionally lecturing upon the subject. After giving a year more to literary pursuits at Bedford Seminary, Mr. Hawxhurst passed four years studying in various schools, and advancing his knowledge of dentistry. He then devoted one year to chemistry and chemical physics, at the State Agricultural College, at Lansing, Michigan. For several years he gave his entire time to dentistry. During this period he joined the Michigan State Dental Association, and helped to organize the Central Michigan Association. He served at different times as Secretary and President of each of these bodies, and a member of various standing committees. He also wrote articles upon different subjects for these Associations, many of which have been published. In the fall of 1873 he entered the State University for the study of medicine, and graduated in 1877. In 1875–76 he took a double course of medicine and dentistry, and graduated in the latter in 1876.

During the past five years he has given his entire attention to dentistry, and has become a member of many dental associations in different States, and has advocated in these and the dental journals, the doctrine that every dentist should found his special studies in his profession upon a thorough medical education. While in Paris, he added fresh stores to his already large stock of information.

In his death the dental profession of Michigan has sustained a severe loss; indeed, the profession of the country can ill afford to lose such men. The Doctor was in the prime of life, and while abroad was preparing himself for a larger field of usefulness, both to his profession and humanity. He was a universal favorite, but was most highly esteemed by those who knew him best. The bereaved wife and mother will have the tender and sincere sympathy of all who knew their lost one.

